# The Effect of Dimethyl Sulfoxide on Supercoiled DNA Relaxation Catalyzed by Type I Topoisomerases

**DOI:** 10.1155/2015/320490

**Published:** 2015-11-22

**Authors:** Bei Lv, Yunjia Dai, Ju Liu, Qiang Zhuge, Dawei Li

**Affiliations:** ^1^Key Laboratory of Forest Genetics and Biotechnology, Nanjing Forestry University, 159 Longpan Road, Nanjing 210037, China; ^2^Division of Chemistry and Biological Chemistry, Nanyang Technological University, 21 Nanyang Link, Singapore 637371; ^3^Collaborative Innovation Center of Sustainable Forestry in Southern China of Jiangsu Province, 159 Longpan Road, Nanjing 210037, China

## Abstract

The effects of dimethyl sulfoxide (DMSO) on supercoiled plasmid DNA relaxation catalyzed by two typical type I topoisomerases were investigated in our studies. It is shown that DMSO in a low concentration (less than 20%, v/v) can induce a dose-related enhancement of the relaxation efficiency of* Escherichia coli* topoisomerase I (type IA). Conversely, obvious inhibitory effect on the activity of* calf thymus* topoisomerase I (type IB) was observed when the same concentration of DMSO is used. In addition, our studies demonstrate that 20% DMSO has an ability to reduce the inhibitory effect on EcTopo I, which was induced by double-stranded oligodeoxyribonucleotides while the same effect cannot be found in the case of CtTopo I. Moreover, our AFM examinations suggested that DMSO can change the conformation of negatively supercoiled plasmid by creating some locally loose regions in DNA molecules. Combining all the lines of evidence, we proposed that DMSO enhanced EcTopo I relaxation activity by (1) increasing the single-stranded DNA regions for the activities of EcTopo I in the early and middle stages of the reaction and (2) preventing the formation of double-stranded DNA-enzyme complex in the later stage, which can elevate the effective concentration of the topoisomerase in the reaction solution.

## 1. Introduction

Topoisomerases are ubiquitous proteins which have the function of manipulation of the topological structures of DNA by generating transient breaks in the double helix in the cells [1–4]. Those nuclear enzymes play vital roles in several cellular processes, such as replication, transcription, and recombination [5–8]. It is reported that some topoisomerases have been selected as the targets of several cancer chemotherapeutic agents [9, 10]. Two types of topoisomerase are classified according to their mechanisms of catalysis: type I topoisomerases change the degree of supercoiling of DNA by causing single-strand breaks and religation, whereas type II topoisomerases cleave both DNA strands at the same time and pass another duplex strand through the break followed by sealing the double-strand break [11, 12]. There are two subclasses of type I enzymes: type IA and type IB.* Escherichia coli* topoisomerase I (EcTopo I), a typical type IA topoisomerase, can only relax DNA with negative supercoiling and require single-stranded DNA regions for its activity [13]. It has been known that EcTopo I can covalently attach to the 5′ end of the broken DNA strand and require divalent metal ions for its catalytic activity [14]. However,* calf thymus* topoisomerase I (CtTopo I) belongs to type IB topoisomerases, which can relax both underwound (negative) and overwound (positive) duplex DNA by formation of a covalent bond with 3′ phosphate [15].

Dimethyl sulfoxide (DMSO), on the other hand, is an important polar aprotic solvent that dissolves both polar and nonpolar compounds. As a result, it has been known as a good solvent for a wide range of organic compounds as well as water, which makes it possible to be used as a powerful tool in chemical and biological researches. Besides its ability to be a good solvent, DMSO has been known as a reagent to enhance the PCR amplification by inhibition of secondary structures in the DNA template or primers, especially in the synthesis of GC-rich gene fragments [16, 17]. Moreover, DMSO has been involved in numerous modified molecular processes such as inhibition of cell proliferation [18], induction of cell differentiation [19], and apoptosis [20].

Since DMSO has an ability to change the topological structures of DNA [21], the effects of DMSO on the efficiency of supercoiling DNA relaxation catalyzed by type I topoisomerases* in vitro* were studied in our lab. Different concentrations of DMSO were used in the pBR322 plasmid relaxation which was catalyzed by EcTopo I (type IA) or CtTopo I (type IB). During the course of further mechanism studies, single-stranded or double-stranded oligonucleotides were employed as the inhibitors of the topoisomerases and the effects of the low concentrations of DMSO were also observed within those systems. In addition, the evidence of topological structure changes caused by DMSO within negative supercoiled plasmid was examined by using atomic force microscopy (AFM). The proposed mechanism of DMSO on enhancing the efficiency of CtTopo I was also discussed here.

## 2. Materials and Methods

### 2.1. Reagents

*Escherichia coli* topoisomerase I was obtained from New England Biolabs (Ipswich, MA).* Calf thymus* topoisomerase I and pBR322 plasmid were provided by Takara Bio Inc. (Shiga, Japan). All the buffers and solutions are prepared by the biological purity water. All the ODNs were purchased from Sigma-Aldrich, which were used as topoisomerase inhibitors here. The sequence of single-stranded ODN (ssODN-1) with 50-mer is 5′ CAACAGCGGTAAGTAGAGCTGGTATTGCACAACATGGATCATGTAACTCG 3′. The double-stranded ODN was prepared by heating the single-stranded ODN (ssODN-1) and its complimentary strand (ssODN-2) in a solution containing 10 mM Tris-HCl (pH = 7.8), 50 mM NaCl, and 1 mM EDTA for 5 minutes at 95°C. The resulting mixture was slowly cooled to room temperature.

### 2.2. Reactions of EcTopo I with Supercoiled pBR322 Plasmid

A 50 *μ*L solution containing 50 mM Potassium Acetate, 20 mM Tris-acetate (pH = 7.9), 10 mM Magnesium Acetate, 100 *μ*g/mL BSA, 200 to 1000 ng pBR322 plasmid, and 0.2 U* Escherichia coli* topoisomerase I was incubated with or without DMSO at 37°C for 0.5 hr. One unit is defined as the amount of enzyme that catalyzes the relaxation of > 95% of 0.5 *μ*g of pUC19 RF I (negatively supercoiled) DNA in 15 minutes at 37°C in a total reaction volume of 25 *μ*L. The obtained products were further analyzed using agarose electrophoresis (1.0%) in the absence of ethidium bromide. The gel was photographed and the DNA bands were measured and quantified using Gel Documentation System (BioRad ChemiDocXRS, US). Percentage of relaxation was defined as the ratio of band density of relaxed DNA over those of relaxed DNA plus supercoiled DNA: relaxed DNA/(relaxed DNA + supercoiled DNA) [22].

### 2.3. Reactions of CtTopo I with Supercoiled pBR322 Plasmid

A 50 *μ*L solution containing 35 mM Tris-HCl (pH = 8), 72 mM KCl, 5 mM MgCl_2_, 5 mM DTT, 5 mM spermidine, 0.1% bovine serum albumin (BSA), 200 to 1000 ng pBR322 plasmid, and 0.5 U* calf thymus* topoisomerase I was incubated with or without DMSO at 37°C for 0.5 hr. One unit is defined as the amount of enzyme that catalyzes the relaxation of 100% of 0.5 *μ*g of pBR322 (negatively supercoiled) DNA in 30 minutes at 37°C in a total reaction volume of 50 *μ*L. The obtained products were further analyzed using agarose electrophoresis (1.0%) in the absence of ethidium bromide. The gel was photographed and the DNA bands were measured and quantified using Gel Documentation System (BioRad ChemiDocXRS, US). Percentage of relaxation was defined as the ratio of band density of relaxed DNA over those of relaxed DNA plus supercoiled DNA: relaxed DNA/(relaxed DNA + supercoiled DNA) [22].

### 2.4. Relaxation Inhibition Assay

One unit of EcTopo I (or CtTopo I) was preincubated with a solution containing 10 nM ODNs and reaction buffers at 37°C for 3 minutes in the presence or absence of 20% DMSO. After that, 1.0 *μ*g supercoiled pBR322 plasmid was added to the abovementioned solution. The final reaction mixture was further incubated at 37°C for 30 minutes. The obtained reaction mixture was analyzed as described above.

### 2.5. Reactions of T7 Endonuclease I with Supercoiled pBR322 Plasmid

A 50 *μ*L solution containing 50 mM NaCl, 10 mM Tris-HCl (pH = 7.9), 10 mM MgCl_2_, 1 mM DTT, 1000 ng pBR322 plasmid, and 0.5 U T7 endonuclease I was incubated with or without DMSO at 37°C for 5 to 60 min. The obtained products were further analyzed using agarose electrophoresis (1.0%) in the absence of ethidium bromide. The gel was photographed and the DNA bands were measured and quantified using Gel Documentation System (BioRad ChemiDocXRS, US).

### 2.6. Experimental Procedures for DNA Sample Preparations and AFM Examination

All micas used in the current studies were modified on their surfaces with (3-aminopropyl)triethoxysilane (APS-micas) following reported procedures [23]. DNA samples for AFM examination were prepared in solutions at first that contained 20 mM Tris-HCl (pH = 7) and 0.1 to 0.01 *μ*g/mL DNA. 5 *μ*L to 10 *μ*L of those DNA solutions was placed next in the middle of the newly prepared APS-mica plates (1 × 1 cm^2^), which were further kept at room temperature for 5 minutes. The surfaces of the APS-mica plates bound by DNA were then rinsed using distilled water for 3 times. AFM images of DNA molecules on the APS-mica plates were obtained in Tapping Mode on a Dimension Edge AFM (Bruker, Santa Barbara, CA) in connection with a Nanoscope VIII controller. Aluminum reflective coating cantilevers with nominal spring constants between 1 and 5 N/m were selected. Scan frequency was 1.9 Hz per line and the modulation amplitude was in a nanometer range. All DNA sample determinations were carried out in air at room temperature.

## 3. Result and Discussion

### 3.1. The Enhancement of Supercoiled pBR322 Relaxation Catalyzed by EcTopo I

**Figure 1:**
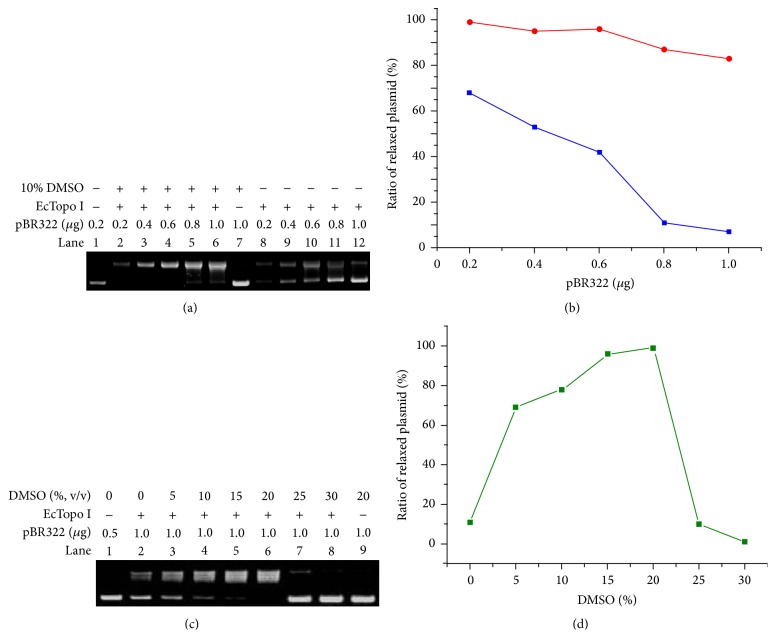
The enhancement of relaxation efficiency of EcTopo I caused by DMSO. (a) Electrophoretic analysis of relaxation products catalyzed by EcTopo I in the presence or absence of 10% (v/v) DMSO. Lane 1: untreated pBR322 plasmid. Lanes 2 to 6: EcTopo I catalyzed relaxation products obtained by incubation of 0.2 *μ*g (Lane 2), 0.4 *μ*g (Lane 3), 0.6 *μ*g (Lane 4), 0.8 *μ*g (Lane 5), and 1.0 *μ*g (Lane 6) pBR322 plasmid in the presence of 10% (v/v) DMSO. Lane 7: the mixture of 1.0 *μ*g pBR322 with 10% (v/v) DMSO. Lanes 8 to 12: EcTopo I catalyzed relaxation products obtained by incubation of 0.2 *μ*g (Lane 2), 0.4 *μ*g (Lane 3), 0.6 *μ*g (Lane 4), 0.8 *μ*g (Lane 5), and 1.0 *μ*g (Lane 6) pBR322 plasmid in the absence of 10% (v/v) DMSO. (b) Correlations between the amount of pBR322 substrate and ratio of relaxed plasmid in the presence (red) and absence (blue) of 10% (v/v) DMSO. (c) Electrophoretic analysis of relaxation products catalyzed by EcTopo I with different concentrations of DMSO. Lane 1: untreated pBR322 plasmid. Lanes 2 to 8: EcTopo I catalyzed relaxation products obtained by incubating 1.0 *μ*g pBR322 plasmid with the concentrations of DMSO of 0% (Lane 2; no DMSO is added), 5% (Lane 3), 10% (Lane 4), 15% (Lane 5), 20% (Lane 6), 25% (Lane 7), and 30% (Lane 8). Lane 9: the mixture of 1.0 *μ*g pBR322 with 20% (v/v). (d) Mutual relations between concentration of DMSO in solution (v/v) and ratio of relaxed pBR322 plasmid.

DMSO was reported to increase the single-stranded regions of negative supercoiled plasmid DNA, which are the crucial binding locations for the activity of type IA topoisomerases. With the aim of exploring whether DMSO can affect the efficiency of plasmid relaxation catalyzed by type IA topoisomerases, 10% DMSO (v/v), optimal condition for synthesis of GC-rich gene fragments [24], was applied to the pBR322 relaxation assay. As shown in Figure 1(a), two bands were observed in each lane of the agarose gel electrophoresis: fast-moving bands (lower bands) were supercoiled plasmid while the slow-moving bands (upper bands) were DNA with relaxed conformation. Without the addition of DMSO, the ratio of relaxed plasmid decreased along with the amount of supercoiled DNA substrate increased as shown in Lanes 8–12 in Figure 1(a). On the other hand, apparent enhancement of the relaxation efficiency can be observed when 10% DMSO (v/v) was added (see Lanes 2–6 in Figure 1(a)).  Figure 1(b) shows the mutual relations between the amount of supercoiled DNA substrates and the ratio of relaxed products obtained by EcTopo I catalyzed reaction in the presence (red) or absence (blue) of 10% (v/v) DMSO, from which an obvious relaxation promotion effect can be observed when DMSO is used.

In order to find out the optimal concentration of DMSO in the EcTopo I catalyzed relaxation reaction, a DMSO dose-dependent examination was conducted during our investigations. As shown in Figure 1(c), an increasing relaxation efficiency was observed with the concentration of DMSO increased from 0% to 20%. The correlation between concentration of DMSO and ratio of relaxed pBR322 plasmid was also described in Figure 1(d). When 20% (v/v) DMSO was used, the maximum relaxation efficiency can be obtained, where 92% of supercoiled pBR322 was relaxed (Lane 6 in Figure 1(c)) while the ratio of relaxed plasmid is only 23% when no DMSO is added (Lane 2 in Figure 1(c)). However, with the concentration of DMSO increased over 20% (v/v), an obvious inhibitory effect was observed and complete inhibition occurred when the concentration of DMSO reached 30%. We speculate that high order protein structures were disintegrated under the DMSO environment with higher concentrations and EcTopo I partially or fully loses its activities accordingly. In addition, a control experiment was also conducted, in which the effect of DMSO on the plasmid substrate was tested. Our result showed that there is no mobility shift difference between pure plasmid (Lane 1 in Figure 1(c)) and a mixture with DNA and 20% DMSO (Lane 9 in Figure 1(c)).

### 3.2. Strongly Inhibitory Effect on the Activities of CtTopo I

**Figure 2:**
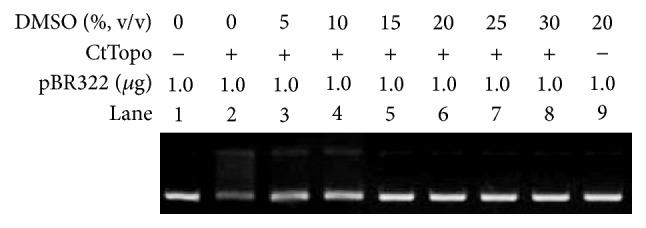
The inhibition of CtTopo I catalyzed relaxation caused by DMSO. Electrophoretic analysis of relaxation products catalyzed by CtTopo I with different concentrations of DMSO. Lane 1: untreated pBR322 plasmid. Lanes 2 to 8: EcTopo I catalyzed relaxation products obtained by incubating 1.0 *μ*g pBR322 plasmid with the concentrations of DMSO of 0% (Lane 2; no DMSO is added), 5% (Lane 3), 10% (Lane 4), 15% (Lane 5), 20% (Lane 6), 25% (Lane 7), and 30% (Lane 8). Lane 9: the mixture of 1.0 *μ*g pBR322 with 20% (v/v).

It has been reported that all type IB topoisomerases share a common fold around the active site region and a common catalytic mechanism [25, 26]. CtTopo I belongs to the family of type IB molecules, which can relax both negatively and positively supercoiled DNA without requiring divalent ions or ATP. By creating the single-stranded break, CtTopo I employ a mechanism called “controlled rotation” to relax supercoiled DNA, where one DNA strand rotates around the other [25]. It is believed that the driven force of the relaxation catalyzed by CtTopo I (type IB) is the torsional strain within the supercoiled DNA molecule. It can be easily predicted that the reaction will not stop until there is no torsional strain to drive the swiveling and DNA is fully relaxed. Therefore, different from EcTopo I (type IA), CtTopo I (type IB) just provide a way to relieve the torsional stress and they do not have to identify directly the global topological structure of DNA. With the aim of investigating whether DMSO can affect the efficiency of plasmid relaxation catalyzed by type IB enzymes, DMSO was used in the pBR322 relaxation assay, where the experiment was conducted in the same way as shown in Figure 1(c) except that EcTopo I was replaced by CtTopo I. Different from EcTopo I, no enhancement of the relaxation efficiency can be observed (Figure 2). On the other hand, a strongly inhibitory effect on CtTopo I can be observed even when the concentration of DMSO is as low as 5%. This happened because CtTopo I do not sense directly the topological structure changes of pBR322 which were induced by DMSO. In addition, CtTopo I are large proteins that contain multiple structural components which may be denatured easily even in a very low concentration of DMSO.

### 3.3. Reduction of Inhibitory Effect on EcTopo I Induced by Double-Stranded Oligodeoxyribonucleotides (dsODNs)

**Figure 3:**
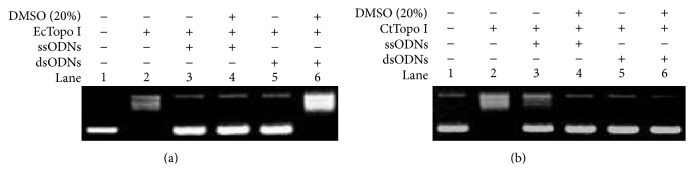
DMSO reduce the inhibition induced by dsODN in EcTopo I catalyzed relaxation. (a) Electrophoretic analysis of EcTopo I catalyzed relaxation inhibition assay in the presence or absence of DMSO. Lane 1: untreated pBR322 plasmid. Lanes 2–6: the mixtures containing EcTopo I (Lane 2); EcTopo I and ssODN (Lane 3); EcTopo I, ssODN, and 20% DMSO (Lane 4); EcTopo I and dsODN (Lane 5); and EcTopo I, dsODN, and 20% DMSO (Lane 6) were preincubated at 37°C for 3 minutes. pBR322 substrate was added to the resulting solutions and incubated at 37°C for 30 minutes. (b) Electrophoretic analysis of CtTopo I catalyzed relaxation inhibition assay in the presence or absence of DMSO. The experiments were conducted in the same ways as shown in (a) except that EcTopo I was replaced by CtTopo I.

The short* oligodeoxyribonucleotides* (ODNs) are powerful tools in the mechanism studies of DNA topoisomerases, where short ODNs were used to simulate some special structures in DNA substrates [27]. It has been reported in the past that eukaryotic topoisomerase I will lose its catalytic activity after it was preincubated with short ODNs. This happens because the short ODNs can form covalent or noncovalent bonds with topoisomerases (ODNs-enzyme complex), which will decrease the effective concentration of the topoisomerases in the reaction solution [28]. The same mechanism studies were also conducted during our investigations. As shown in Figure 3(a), both of the relaxations were inhibited completely after EcTopo I were preincubated with single-stranded ODNs (Lane 3 in Figure 3(a)) and double-stranded ODNs (Lane 5 in Figure 3(a)). With the purpose of studying whether DMSO can affect the inhibition caused by ONDs, 20% DMSO was used. As shown in Figure 3(a), the inhibitory effect induced by ssODNs cannot be removed when 20% DMSO was added (Lane 4 in Figure 3(a)). Conversely, an obvious promotion (Lane 6 in Figure 3(a)) was observed when 20% DMSO was added to the relaxation reaction which was originally inhibited by dsODNs. We speculated that DMSO has a function of preventing the formation of dsODNs-enzyme complex to increase the effective concentration of EcTopo I in the reaction solution. However, EcTopo I may exhibit higher affinity to ssODNs, which cannot be separated by DMSO. The result shown here corresponds with the established mechanism of EcTopo I, where type IA enzymes preferentially bind to the ssDNA region of the supercoiled plasmid [13]. In addition, the same experiments were conducted except that EcTopo I was replaced by CtTopo I (Figure 3(b)). Our results showed that neither ssODN nor dsODNs caused inhibitory effects can be removed by 20% DMSO. Type IB enzyme was reported to encircle the duplex DNA by forming a clamp around it [25, 29], which indicated that DMSO may not separate the ODNs-enzyme complex easily. In addition, DMSO itself has a much stronger inhibitory effect on the activity of CtTopo I even when the ODNs-enzyme complex can be removed.

### 3.4. AFM Examination of the Topological Structure Changes of the Supercoiled Plasmid Treated with DMSO

**Figure 4:**
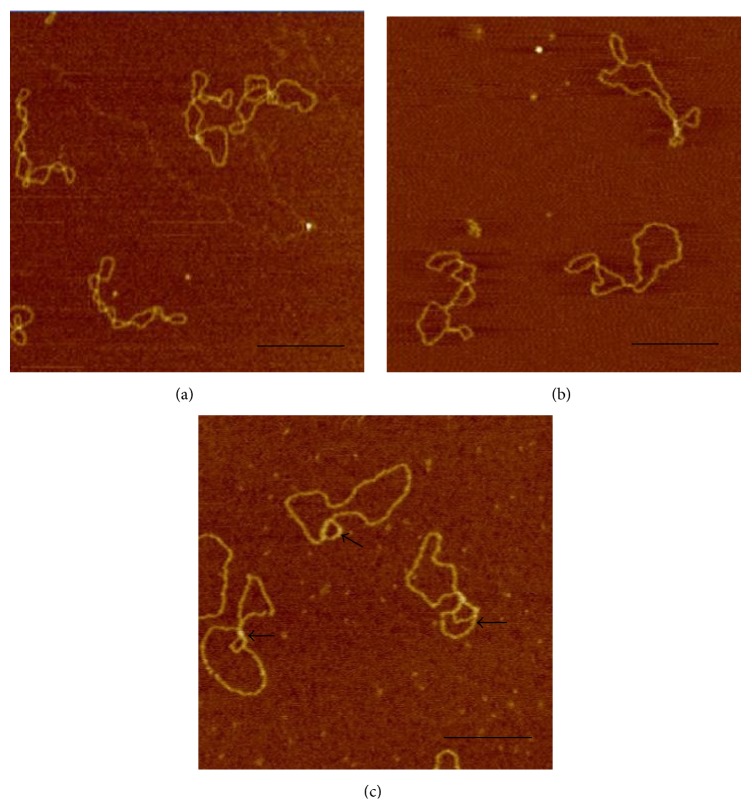
AFM image of negatively supercoiled pBR322 in the different concentrations of DMSO. (a) Untreated pBR322. (b) pBR322 in 5% DMSO. (c) pBR322 in 10% DMSO. The black arrows indicate the single-stranded regions in plasmid.

Atomic force microscopy (AFM) has been known to be a powerful tool for determining certain subtle alternations in DNA topological features [23, 30, 31]. With the aim of examining whether DMSO can indeed change the topological structure of negative supercoiled plasmid, AFM was used for the first time to test the structural alternation of pBR322 caused by DMSO during our investigations. For the purpose of comparison, the pure negatively supercoiled plasmid was examined firstly. As shown in Figure 4(a), a compact supercoiled structure can be observed. It is known, on the other hand, that the entire topological structure of plasmid will be changed if there are some locally loose regions caused by the environment [17]. The negatively supercoiled pBR322 was consequently incubated next with 5% DMSO during our examinations. As shown in Figure 4(b), the topological molecular skeleton became loose comparing with the pure negatively supercoiled DNA molecules shown in Figure 4(a). We speculated that this happens because some locally loose areas were created by DMSO environment and the other parts of DNA become more twisted, which will lead to the change of the writhe number of negative supercoiled plasmid according to the “DNA topological conservation law” [8]. We believe that the reason why the locally loose (or single-stranded) regions cannot be observed in Figure 4(b) is that the areas are too small to be identified and they are beyond the resolution limitations of our AFM. In order to directly observe the locally loose areas created by DMSO, we decided to increase the concentration of DMSO. As anticipated, some single-stranded regions were observed when 10% (v/v) DMSO was used. When the concentration of DMSO was increased to 20% (v/v), a lot of noise appeared and no DNA molecule can be found in the AFM images (data not shown).

In addition, most of the molecules bands are negatively supercoiled (Lane 7 in Figure 1(a)), which is contrary to the results shown in Figure 4(c). This happens because the negatively supercoiled pBR322 DNA molecules are very sensitive to the concentration of DMSO and the condition in agarose gel running system is quite different from those DNA samples in the tubes. Therefore, the topological conformations of DNA molecules were changed in agarose gel, which was caused by the dilution of the concentration of DMSO in the gel.

### 3.5. The Enhancement of Reaction Efficiency of T7 Endonuclease I Caused by DMSO

**Figure 5:**
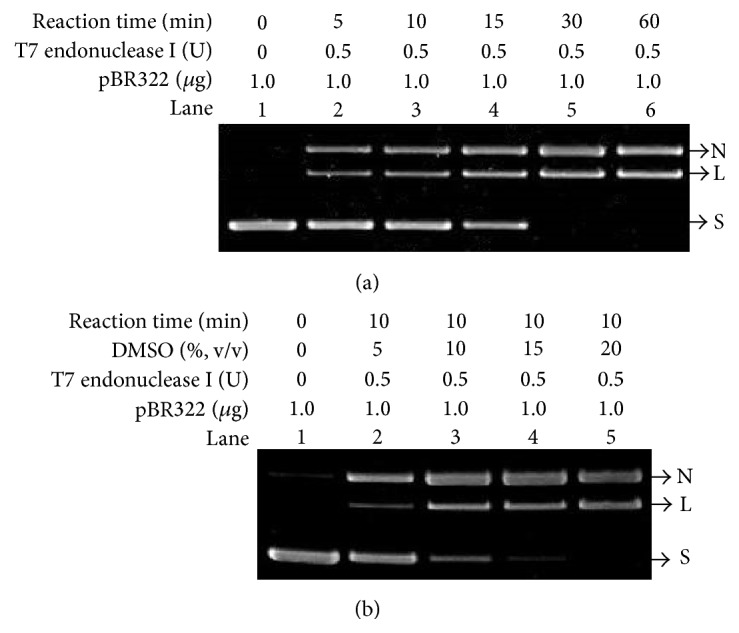
The enhancement of reaction efficiency of T7 endonuclease I caused by DMSO. S: supercoiled plasmid; L: linear DNA; N: nicked plasmid. (a) Electrophoretic analysis of products catalyzed by T7 endonuclease I in the absence of DMSO. Lane 1: untreated pBR322 plasmid. Lanes 2 to 6: T7 endonuclease I catalyzed reaction products obtained by incubating negatively supercoiled pBR322 with T7 endonuclease I at 37°C for 5 min (Lane 2), 10 min (Lane 3), 15 min (Lane 4), 30 min (Lane 5), and 60 min (Lane 6). (b) Electrophoretic analysis of products catalyzed by T7 endonuclease I in the presence of DMSO. T7 endonuclease I catalyzed reaction products obtained by incubating 1.0 *μ*g pBR322 plasmid with the concentrations of DMSO of 5% (Lane 2), 10% (Lane 3), 15% (Lane 4), and 20% (Lane 5) at 37°C for 10 min.

To further confirm that DMSO can indeed increase single-stranded characteristics in DNA, T7 endonuclease I, a type of endonuclease that cleaves non-perfectly matched DNA, was used in our studies. Since the sequence of pBR322 DNA contains an inverted repeated region, a cruciform structure can be formed within the negatively supercoiled pBR322 [32, 33]. For the comparison purpose, negatively supercoiled pBR322 was first incubated with T7 endonuclease I in buffer solution that contains no DMSO for different reaction time. As shown in Figure 5(a), two new bands were observed. The upper band is plasmid with nicked form and the lower band is linear DNA. With the aim of investigating whether DMSO can indeed increase single-stranded characteristics in DNA, DMSO was added to the buffer solution and the reaction mixture was incubated at 37°C for 10 min (Figure 5(b)), a condition that can convert about half of the supercoiled plasmid into nicked or linear form without DMSO (Lane 3 in Figure 5(a)). As anticipated, all supercoiled DNA were cleaved when 20% DMSO was used (Figure 5(b)), which indicated that DMSO can significantly promote the reaction efficiency by increasing the single-stranded regions of the plasmid. The result shown here is consistent with the discussion in Figures 1–4. In addition, supercoil-induced formation of cruciform structure is greatly influenced by the salts and temperature [34]. The conditions used in our AFM studies do not favor the formation of cruciform structure and no such structure was observed (Figure 4).

### 3.6. The Proposed Mechanism for DMSO Elevating EcTopo I Catalyzed DNA Relaxation Efficiency

Combining all the lines of evidence shown above, we proposed a possible mechanism for DMSO elevating EcTopo I catalyzed DNA relaxation efficiency. Since the double helix is underwound in the negatively supercoiled plasmid, the single-stranded characters can be found. In the early stage of the reaction, an equilibrium situation occurs between the plasmid with negative supercoiling and DNA molecules with some single-stranded regions in a certain buffer and temperature condition. Once DMSO is added, more locally loose regions will be created, which are the crucial binding areas for activity of CtTopo I. It has been established that type I enzymes alter linking number in steps of one, removing one supercoil (i.e., one turn of the helix) at a time [35]. This means that the enzyme must recognize and bind to the active sites when the new round of catalysis begins each time. All the supercoiled substrates have been partly relaxed at the middle stage. However, with the negative supercoil decreased, the single-stranded character has been lower. At this moment, DMSO can also help to increase some locally loose region for the active binding of EcTopo I. We therefore believed that the increasing of the single-stranded DNA regions induced by DMSO in the early and middle stages of the reaction is the crucial reason why the relaxation was stimulated. On the other hand, it has been proven in the past that the relaxation efficiency of EcTopo I decreased significantly as the linking number of the plasmid increased to near the linking number of fully relaxed DNA molecules [35]. This happens because the character of helical structure of plasmid in the later stage of the reaction is very similar to those of linear duplex DNA, which may decrease the possibility to form the single-stranded binding sites for enzymes. More importantly, the formed duplex regions with the characters of linear DNA within the plasmid will be exposed to the free enzymes in the solution and act as the inhibitor by forming the duplex-enzyme complex. As discussed in Figure 3(a), the DMSO environment can help to prevent the formation of dsODN-enzyme complex. We therefore speculated that low concentration of DMSO may play an important role in keeping the effective concentration of EcTopo I by preventing the formation of duplex-enzyme complex in the later stage of the reaction.

## 4. Conclusion

DMSO has been widely used in chemical and biological studies. It was known to have the ability to modulate a lot of molecular processes including replication, regulation of gene expression, recombination, and PCR* in vitro* [16, 17]. It has been studied that the structure changes of DNA substrate and enzyme caused by DMSO were the crucial reasons for those modified biological processes [18–20]. The effect of DMSO on the plasmid relaxation reaction catalyzed by EcTopo I and CtTopo I was reported here through using gel electrophoresis and AFM for the first time. We speculated that DMSO can improve the relaxation efficiency of EcTopo I by creating more single-stranded regions in the early and middle stage and preventing formation of duplex-enzyme complex in the later stage of the reaction. It is our hope that the information presented in this report could benefit our understanding of the mechanism of type I enzyme catalyzed DNA relaxation. The results reported here may also shed new light on the general mechanism in some DMSO modified DNA transactions and cellular processes.
